# Repression of *MicroRNA-124-3p* Alleviates High-Fat Diet–Induced Hepatosteatosis by Targeting Pref-1

**DOI:** 10.3389/fendo.2020.589994

**Published:** 2020-11-26

**Authors:** Guan Wang, Haibo Zou, Chunyou Lai, Xiaolun Huang, Yutong Yao, Guangming Xiang

**Affiliations:** Department of Hepatobiliary and Pancreatic Surgery, Cell Transplantation Center, Sichuan Academy of Medical Sciences, Sichuan Provincial People’s Hospital, School of Medicine, University of Electronic Science and Technology of China, Chengdu, China

**Keywords:** nonalcoholic fatty liver disease, high-fat diet, *miR-124-3p*, Pref-1, miRNA

## Abstract

Nonalcoholic fatty liver disease (NAFLD) is the common disease in the liver, which is associated with metabolic syndrome and hepatocellular carcinoma. Accumulated evidence establishes that small non-coding microRNAs (miRNAs) contribute to the initiation and progression of NAFLD. However, the molecular repertoire of miRNA in NAFLD is still largely unknown. Here, using an integrative approach spanning bioinformatic analysis and functional approaches, we demonstrate that *miR-124-3p* participates in the development of NAFLD by directly targeting preadipocyte factor-1 (Pref-1). In response to high-fat diet (HFD), expression of *miR-124-3p* was increased in the liver. Inhibition of *miR-124-3p* expression led to a dramatic reduction of triglyceride contents in hepatocytes, in parallel with decreased inflammatory factors. Mechanistically, *miR-124-3p* directly controls the transcription of Pref-1, a secretory factor that has been proved to resist metabolic syndrome. Our work identifies a novel molecular axis in hepatosteatosis, and highlights *miR-124-3p*/Pref-1 as potential targets for clinical interventions of NAFLD.

## Introduction

Non-alcoholic fatty liver disease (NAFLD) is the most common liver disease, which is characterized by excessive hepatic fat deposition and a wide range of pathologies encompassing steatohepatitis, fibrosis, cirrhosis, liver failure, and hepatocellular carcinoma (1, 2). A number of molecular abnormalities that occur in hepatosteatosis confer to the initiation and progression of NAFLD. Evidence from experimental and clinical studies suggests a complicated interplay of multiple biological processes in the disease development, including obesity, dysbiosis of the intestinal microbiome, heightened intestinal barrier permeability, metabolic endotoxemia and inflammations (3–5). Despite the high prevalence and clinical importance, factors leading to NAFLD were still poorly understood and few efficacious therapies exist (6). Therefore, molecular understandings of the initiation and progression of NAFLD are urgently needed to provide a basis for therapeutic design.

MicroRNAs (miRNAs) are small non-coding RNAs with 18~24 nucleotides, which regulates gene expression by binding to mRNAs and impairs the process of translation (7). MiRNAs are emerging as new regulators of glucose and lipid metabolism during liver development and disease progression, including NAFLD (8, 9). For example, hepatic *miR-122* affects gene expressions in cholesterol and lipid metabolism, and thereby maintains liver homeostasis (10, 11). *miR-27a* attenuated hepatic *de novo* lipogenesis and alleviated obesity-initiated NAFLD by inhibiting *Fasn* and *Scd1* in liver (12). *miR-375* is up-regulated in NASH patients, and its inhibition ameliorates lipid accumulation and decreases inflammatory cytokines (13, 14). Despite these studies, however, the molecular repertoire of miRNA in NAFLD is still largely unknown.

In this study, we reveal a novel function of *miR-124-3p* in hepatic lipid metabolism. *MiR-124-3p* expression in the liver is increased under high-fat diet (HFD). Inhibition of *MiR-124-3p* reduces triglyceride contents as well as inflammatory factors in hepatocytes, and vice versa. Mechanistically, *miR-124-3p* directly controls the transcription of Pref-1, a secretory factor that resists metabolic syndrome. Our work establishes the novel function of *miR-124-3p* in maintaining liver homeostasis, and defines *miR-124-3p* as a potential target for clinical interventions of NAFLD.

## Materials and Methods

### Animals and Cell Culture

C57BL/6J mice were obtained and raised in the animal center of Sichuan Academy of Medical Science. Experimental mice were kept in SPF conditions with standard housing conditions in a temperature-controlled environment with 12-h light/dark cycles and received chow diet (CD) or high-fat diet (HFD, from 8-week age) and water *ad libitum* for 12 weeks (15). Animals studies were conducted in accordance with institutional guidelines. For primary hepatocyte culture, we conducted a two-step collagenase perfusion method as described (16). Isolated hepatocytes were cultured in Williams E medium (Sigma) including HepExtend™ Supplement (Gibco), plus penicillin/streptomycin and gentamycin in 37°C under 5% CO_2_.

### Oil Red O Staining

For oil red O staining to validate the hepatosteatosis in mice with HFD, fresh liver samples were fixed in 4% paraformaldehyde (PFA) solution, and embedded with OCT for subsequent staining with Oil Red O, according to standard protocols (17).

### MicroRNA Assay and Analysis

To detect the profile of miRNAs in mouse livers under HFD and CD, a total of miRNAs were extracted using miRNeasy mini kit (QIAGEN). The microarray hybridization of miRNAs was performed using GeneChip miRNA 3.0 Array (Affymetrix). Microarray datasets were presented in volcano plot and GO biological process classification, and miRNAs with 2-fold or greater fold change with P-value *<* 0.05 were considered differentially expressed.

### Plasmid Construct and Luciferase Reporter Assays

To confirm that *miR-124-3p* directly targets Pref-1, a luciferase reporter assay was performed. Briefly, the pmirGL-control luciferase vector, pmirGLO dual-Luciferase miRNA target expression vector and luciferase reporter assay system were purchased from Youbia (China). The pmirGLO-control vector was used to construct pGL-pref-1, which contained the 3’UTR of mouse pref-1 (Genbank accession NM003836). DNA fragment of Pref-1 was constructed into vector (18). For luciferase reporter assay, control vector or *miR-124-3p* plasmid and reporter plasmids were co-transfected into cells. Luciferase activities were measured at 48 h post-transfection by using the dual-luciferase assay system (Turner BioSystem, USA).

### Protein Lysis and Western Blots

For western blots, cultured cells were lysed in 2% SDS buffer plus protease and phosphatase inhibitors (Thermo Scientific, USA). The lysates of equivalent total proteins were separated on SDS-PAGE. Then proteins were transferred into PVDF membrane (Millipore) for incubation with primary/secondary antibodies. The primary antibodies used were as follows, Pref-1 (Cell Signaling Technology, Cat#2069) and GAPDH (Cell Signaling Technology, Cat#2118).

### RNA Extraction and qPCR

Total RNA was extracted by Animal miRNA Isolation Kit (Foregene, China) and Cell Total RNA Isolation Kit (Foregene, China) followed by standard procedure. The PrimeScriptTM RT reagent Kit (Takara) was used to reverse transcript to cDNA, and SYBR Premix Ex TaqTM II (Takara) was used to qPCR assay in Bio-rad system. The qPCR primers were list as follows: *miR-124-3p*, F: 5’-TAAGGCACGCGGTGAATGCC-3’, R: 5’-GATTGAATCGAGCACCAGTTAC-3’; TNFα, F: 5’-CAGGCGGTGCCTATGTCTC-3’, R: 5’-CGATCACCCCGAAGTTCAGTAG-3’; Pref-1, F: 5’-TTCGGCCACAGCACCTATG-3’, R: 5’-GGGGCAGTTACACACTTGTCA-3’.

### RNA Interference

For Pref-1 knockdown, targeted siRNA were transfected into cells using Lipofectamine RNAiMAX reagent (Invitrogen, USA) in Opti-MEM (Invitrogen, USA) over 72 h. The siRNA sequences were as follows, siRNA negative-control: sense-UCCGGAACUGUUACGUGAA; antisense-UUCACGUAACAGUUCCGGA. siRNA Pref-1: sense: UCCUGAAGGUGUCCAUGAA; antisense: UUCAUGGACACCUUCAGGA.

### Statistical Analysis

Statistical analysis was performed in GraphPad Prism. Data were expressed as mean ± SD from at least three independent experiments. Statistical differences between two groups were analyzed by Student’s *t* test, and multi-group comparisons by one-way ANOVA followed by Tukey *post hoc* tests. P<0.05 was considered statistically significant; **indicates p<0.01, and *** p<0.001.

## Results

### MiR-124-3p Expression Is Increased by HFD-Induced Hepatosteatosis

**Figure 1:**
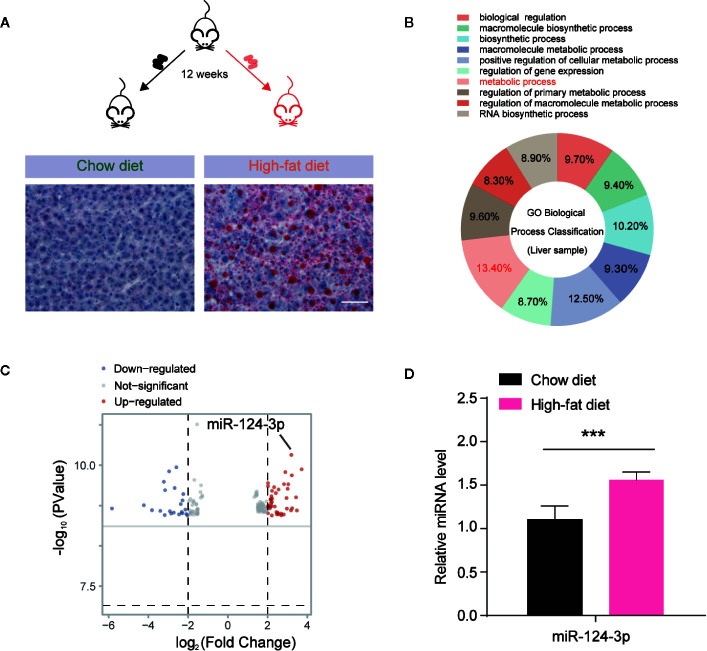
*MiR-124-3p* expression is increased by high-fat diet (HFD)-induced hepatosteatosis. **(A)** A schematic model illustrating that mice fed with chow diet (CD) and high fat diet (HFD) for 12 weeks, and hepatosteatosis were examined by Oil Red O staining. Scale bar 100 μm. **(B)** A diagram showing that metabolic process are relevant to miRNA expression analyzed by GO biological classification in the liver of HFD mice compared to CD controls. **(C)** Volcano Plots showing the different miRNA expression in the liver of HFD mice compared to CD controls. The arrow indicates *miR-124-3p*. **(D)** qPCR results showing that *miR-124-3p* is upregulated in the liver of HFD mice compared to CD controls. Results were averages of three independent experiments. Data represent mean ± SD. ***P < 0.001.

To investigate the miRNAs involved in hepatosteatosis, we assessed the hepatic miRNA profile in C57BL/6J mice fed with high fat diet (HFD) for 12 weeks, in comparison to littermate controls fed with chow diet (CD). Then we extracted the total miRNA from mouse livers and performed microarray hybridization using GeneChip miRNA 3.0 Array (Affymetrix). Histochemical results confirmed the lipid accumulation by Oil Red O staining in the liver of HFD mice (**Figure 1A**). By miRNA screening, we found that altered miRNAs between HFD and CD mice were enriched in metabolic process by GO (Gene Ontology) biological process classification (**Figure 1B**). As shown in the volcano plot, we differentiated the increased and decreased miRNAs. Particularly, it’s found that *miR-124-3p* was dramatically increased in the liver of HFD mice (**Figure 1C**). And this increasing was further validated by quantitative evaluation from more paired HFD/CD mice (**Figure 1D**). All these data suggests that increased *miR-124-3p* may play a vital role in HFD-induced hepatosteatosis.

### MiR-124-3p Positively Regulates Lipid Contents in Hepatocytes

**Figure 2:**
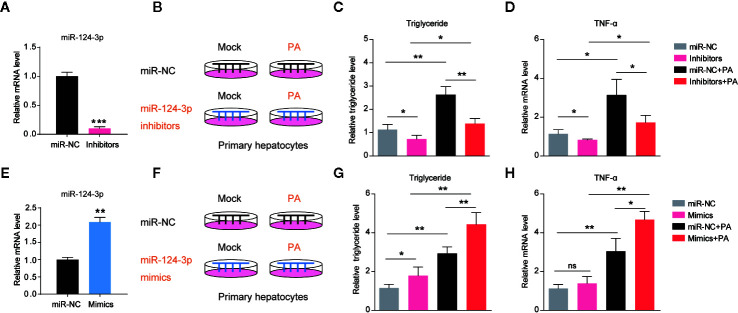
*MiR-124-3p positively regulates lipid contents in hepatocytes.* **(A)** qPCR results showing that *miR-124-3p* inhibitors decreased endogenous *miR-124-3p* level in primary hepatocytes. **(B–D)** A schematic model **(B)** illustrating that primary hepatocytes were treated with palmetric acid (PA) with or without *miR-124-3p* inhibitors. Biochemical results **(C)** showing that *miR-124-3p* inhibition decreased PA-induced triglyceride in hepatocytes. qPCR results **(D)** showing that *miR-124-3p* inhibition decreased PA-induced TNF-α in hepatocytes. **(E)** qPCR results showing that *miR-124-3p* mimics increased *miR-124-3p* level in primary hepatocytes. **(F–H)** A schematic model **(F)** illustrating that primary hepatocytes were treated with palmetric acid (PA) with or without *miR-124-3p* mimics. Biochemical results **(G)** showing that *miR-124-3p* overexpression increased PA-induced triglyceride in hepatocytes. qPCR results **(H)** showing that *miR-124-3p* overexpression increased PA-induced TNF-α in hepatocytes. Results were averages of three independent experiments. Data represent mean ± SD. *P < 0.05, **P < 0.01 and ***P < 0.001.

Based on the finding that *miR-124-3p* was induced by HFD in mouse liver, we asked whether induction of *miR-124-3p* has a causal effect on lipid accumulation. Therefore, we examined the lipid contents in hepatocytes after *miR-124-3p* inhibition. As a first step, we knocked down *miR-124-3p* expression by transfection of its inhibitors in primary hepatocytes, and the transfection efficiency was confirmed by RT-qPCR (**Figure 2A**). Next, we inactivated *miR-124-3p* expression in palmetric acid (PA) treated hepatocytes (**Figure 2B**). Although PA could increase triglycerides in control hepatocytes (19), however, *miR-124-3p* inhibitors dramatically reduced triglyceride contents by PA treatment (**Figure 2C**). Excessive lipid accumulation often coincides with increased inflammation (20). We found that *miR-124-3p* inhibitors could also decrease the level of TNFα by PA treatment, indicating that *miR-124-3p* inactivation ameliorates liver inflammation (**Figure 2D**). Reciprocally, we examined whether overexpression of *miR-124-3p* by its mimics is sufficient to increase lipid contents and inflammations in hepatocytes. Results showed that *miR-124-3p* overexpression increased triglyceride levels and TNFα expression, suggesting that *miR-124-3p* may have a causal effect on steatosis in the liver (**Figures 2E–H**). Taken together, these results suggest that *miR-124-3p* positively regulates lipid contents and inflammation in hepatocytes.

### MiR-124-3p Directly Targets Pref-1 in Hepatocytes

**Figure 3:**
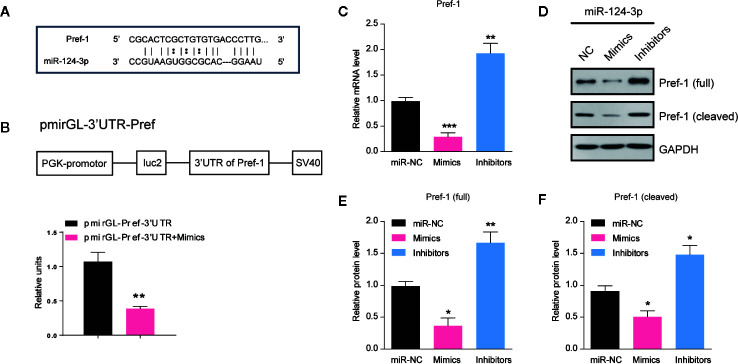
*MiR-124-3p* directly targets Pref-1 in hepatocytes. **(A)** Sequence analysis of *miR-124-3p* binding with the 3’-UTR of Pref-1. **(B)** A schematic diagram showing the pmirGL-3’UTR Pref-1 vector, and luciferase activities were measured by the luciferase reporter assay. **(C)** qPCR results showing the mRNA levels of Pref-1 in hepatocytes transfected with *miR-124-3p* mimics, inhibitors or scramble sequence. **(D, E)** Western blots **(D)** and quantifications **(E, F)** showing the protein levels of full-length and cleaved Pref-1 in hepatocytes transfected with *miR-124-3p* mimics, inhibitors or scramble sequence. Results were averages of three independent experiments. Data represent mean ± SD. *P < 0.05, **P < 0.01 and ***P < 0.001.

To clarify how *miR-124-3p* participates in the regulation of lipid metabolism, we next examined the potential targets of *miR-124-3p*. By sequence analysis, we found that Preadipocyte factor 1 (Pref-1) was a potential candidate of *miR-124-3p* in the liver (**Figure 3A**). Pref-1 is a transmembrane protein that could be cleaved at the extracellular domain to generate a soluble form that reduces hepatosteatosis and hyperglycemia (21, 22). To confirm that *miR-124-3p* directly targets Pref-1, we performed a luciferase reporter assay. Data showed that the activity of the reporter plasmid with *miR-124-3p* mimic was decreased, indicating that *miR-124-3p* binds to the 3’-UTR of Pref-1 and thus regulates Pref-1 expression (**Figure 3B**). Results of qPCR showed that the mRNA level of Pref-1 was decreased by *miR-124-3p* overexpression and increased by its knockdown (**Figure 3C**). Consistently, the protein levels of both full and cleaved Pref-1 were both negatively regulated by *miR-124-3p* (**Figures 3D–F**). Therefore, we propose that *miR-124-3p* directly targets Pref-1 to regulate its expression.

### miR-124-3p Regulates Lipid Contents via Pref-1 in Hepatocytes

**Figure 4:**
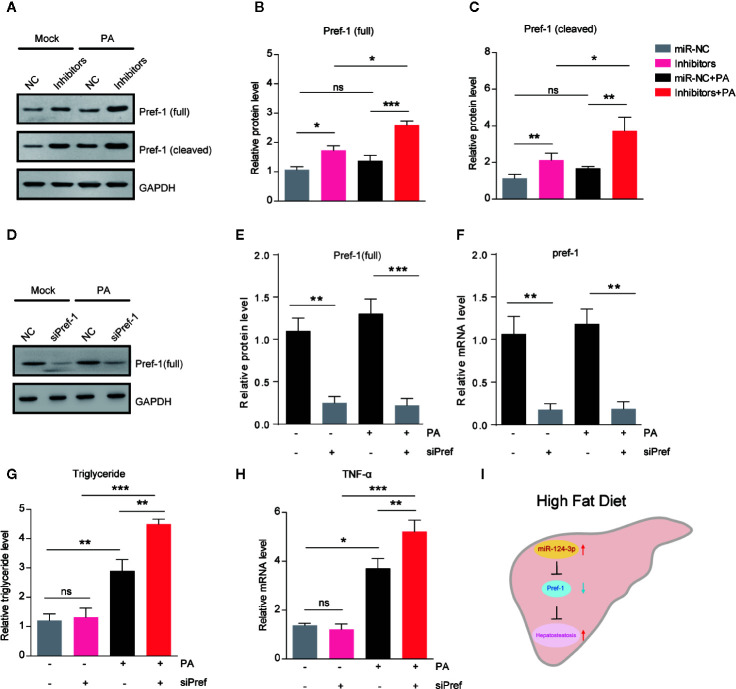
*MiR-124-3p regulates lipid metabolism via Pref-1 in hepatocytes.* **(A–C)** Western blots **(A)** and quantifications **(B, C)** showing the protein levels of full-length and cleaved Pref-1 in hepatocytes transfected with *miR-124-3p* inhibitors or scramble sequence and treated with palmetric acid (PA). Results were averages of three independent experiments. **(D–F)** Western blots and qPCR showing the decreased protein and mRNA level of Pref-1 in cells transfected with siPref-1. **(G)** Biochemical results showing that siPref-1 increased PA-induced triglyceride. **(H)** qPCR results showing that siPref-1 increased PA-induced TNF-α expression. **(I)** A schematic model highlighting the role of *miR-124-3p* alleviates hepatosteatosis by directly targeting Pref-1 in hepatocytes under lipid stress. Data represent mean ± SD. *P < 0.05, **P < 0.01 and ***P < 0.001.

Pref-1 has been proved to resist HFD–induced hepatosteatosis and obesity (23, 24). To study whether *miR-124-3p* regulates hepatic lipid metabolism through Pref-1, we assessed the changes of Pref-1 after PA treatment in *miR-124-3p* inactivated hepatocytes. Western blot results showed that *miR-124-3p* inhibitors could induce expression of full and cleaved Pref-1 with or without PA (**Figures 4A–C**). It’s noted that Pref-1 expression was slightly increased by PA, however, dramatically increased by *miR-124-3p* inhibitors (**Figures 4A–C**), suggesting that *miR-124-3p* inactivation was necessary for Pref-1 expression under lipid stress conditions.

To strengthen the point that *miR-124-3p* regulates hepatosteatosis through Pref-1, we examined the effect of Pref-1 knockdown on the lipid metabolism and inflammation in hepatocytes (**Figures 4D–F**). Results showed that Pref-1 knockdown did not dramatically alter triglyceride levels or TNFα expression under basal condition. However, in the presence of PA, Pref-1 knockdown caused a more dramatic increasing of triglyceride levels or TNFα expression, indicating that Pref-1 is required for the resistance of triglyceride accumulation under lipid stress (**Figures 4G–H**). Thus, we conclude that *miR-124-3p* regulated Pref-1 expression is critical for the maintenance of lipid homeostasis in hepatocytes.

## Discussion

Altered miRNA expression in response to genetic/epigenetic factors or environmental conditions confers to the onset of NAFLD (25). However, the molecular repertoire of miRNA in the initiation and progression of NAFLD is largely unknown. The present study demonstrates that *miR-124-3p* participates in hepatosteatosis in response to lipid stress. Inhibition of *miR-124-3p* in hepatocytes promotes Pref-1 expression and thus ameliorates lipid accumulation (**Figure 4I**).

In decades, numerous miRNAs were identified to regulate a wide spectrum of metabolic processes, including lipid homeostasis, glucose catabolism, and inflammation, which were known to be epigenetically deregulated in NAFLD (26). Altered hepatic miRNA profile has been described in both in humans and animal models of NAFLD (27). A recent study assessed circulating miRNAs in NASH patients and found that among 84 circulating miRNAs (13). For example, *miR-122* and *miR-192* were significantly upregulated and others were downregulated. Comparing to these studies, we revealed that *miR-124-3p* was particularly increased in the liver of HFD mice. This increasing was consistent with our finding that PA treatment could induce *miR-124-3p* expression in primary hepatocytes. Therefore, we conclude that *miR-124-3p* expression is dynamically regulated by lipid stress *in vitro* and *in vivo*. Moreover, we revealed that increased *miR-124-3p* expression may have a causal effect on the lipid accumulation in hepatocytes. Our data showed that inhibition of *miR-124-3p* decreased PA-induced triglyceride levels, and particularly, overexpression of *miR-124-3p* is sufficient to increase triglyceride level (**Figure 2G**), which is more pronounced after PA treatment. Thus, *miR-124-3p* is a critical regulator in lipid homeostasis of the liver. The impact of *miR-124-3p* on lipid metabolism indicates its potential therapeutic implication of NAFLD.

*MiR-124-3p* is a widely expressed miRNA in mammalian cells, and previous studies reveal its biological function in tumorigenesis and neural diseases. For example, *miR-124-3p* regulates aerobic glycolysis and induces chemoresistance in glioma cells through AMPK pathway (28). *miR-124-3p* also represses the migration and invasion of bladder cancer cells *via* ROCK1 (29). In the liver, *miR-124-3p* modulates autophagy through Beclin and LC3 and thus participates in hepatic impact injury (30). However, the role of *miR-124-3p* in metabolic diseases is not yet well understood. A recent study showed that *miR-124* represses genes associated with fatty acid and triglyceride breakdown, and thus promotes triglyceride accumulation in hepatoma cells (31). Consistent with this finding, our study demonstrate that *miR-124-3p* has a direct effect in the liver under lipid stress conditions. We further revealed that *miR-124-3p* directly targets Pref-1 that resists hepatic lipid accumulation. Pref-1 belongs to the NOTCH family of epidermal growth factor-like repeat-containing proteins, which can inhibit adipogenesis and resist to high fat diet-induced obesity in mice (21, 32). In this study, we identified that *miR-124-3p* negatively regulates the expression of Pref-1 and its soluble fragment. It’s noticed that *miR-124-3p* overexpression could induce a slight but reproducible triglyceride increasing in hepatocytes under basal condition (**Figure 2G**). However, Pref-1 knockdown alone failed to produce a similar effect like *miR-124-3p* overexpression (**Figure 4G**), suggesting that *miR-124-3p* may regulate lipid metabolism partly through Pref-1 independent mechanisms. Interestingly, after PA treatment, Pref-1 knockdown phenocopies the effect of *miR-124-3p* overexpression, indicating that *miR-124-3p*-controlled lipid accumulation is largely dependent on Pref-1 under lipid stress condition. Nevertheless, our study at least reveals that *miR-124-3p*/Pref-1 is a novel molecular axis in hepatosteatosis. Future studies would be directed at the assessment of *miR-124-3p*/Pref-1 axis in resistance to hepatosteatosis in animal models and clinical trials.

## Conclusion

In summary, our study identifies a novel function of *miR-124-3p* in the liver. Hepatocytes with *miR-124-3p* knockdown have decreased triglyceride contents and inflammatory cytokines. This work provides a therapeutic target for potential interventions.

## Data Availability Statement

The raw data supporting the conclusions of this article will be made available by the authors, without undue reservation.

## Ethics Statement

The animal study was reviewed and approved by Sichuan Provincial People’s Hospital.

## Author Contributions

GW, YY, and GX designed the study and wrote the manuscript. GW, HZ, CL, XH, YY, and GX performed the experiments and analyzed the data. All authors contributed to the article and approved the submitted version.

## Funding

This work was supported by grants from Specialized Fund for Basic Scientific Research, Sichuan Provincial Science and Technology Department (2018YSKY0017).

## Conflict of Interest

The authors declare that the research was conducted in the absence of any commercial or financial relationships that could be construed as a potential conflict of interest.
